# Microbial Diversity, Nutritional Composition, and Health Implications of Fermented Locust Bean Seed (Dawadawa) From Ghana

**DOI:** 10.1155/ijfo/8444101

**Published:** 2025-09-19

**Authors:** Kwaku Moses Golly, Emmanuel Tetteh Doku, Afia Sakyiwaa Amponsah, Patience Kyei, Comfort Gyadua

**Affiliations:** ^1^ Department of Hospitality and Tourism, Sunyani Technical University, Sunyani, Bono Region, Ghana, stu.edu.gh; ^2^ Department of Pharmaceutical Sciences, Sunyani Technical University, Sunyani, Bono Region, Ghana, stu.edu.gh

**Keywords:** condiment, indigenous fermented food, microbiome, proximate composition

## Abstract

Fermented foods play a vital role in global nutrition, and dawadawa, a traditional African locust bean (*Parkia biglobosa*) fermentation product, is a key dietary component in Northern Ghana. This study investigates the microbial diversity, nutritional composition, and health implications of dawadawa produced across six major communities. The determination of nutritional composition and bacterial communities in dawadawa was conducted using standard spectrometric methods and 16S RNA sequencing, respectively. Proximate analysis revealed significant variations in fat (17.45%–27.70%), protein (36.12%–50.00%), and fiber (6.39%–7.32%) across different locations, with Sunyani samples exhibiting the highest protein content. Mineral analysis showed notable differences in iron (79.60–135.00 mg/kg), zinc (37.75–91.77 mg/kg), and calcium (0.73%–1.61%), suggesting potential nutritional benefits. Microbial profiling using 16S rRNA sequencing identified *Bacillus*, *Staphylococcus*, *Streptococcus*, and *Lactobacillus* as predominant genera, with *Bacillus subtilis* being the most abundant species. Domestic dawadawa exhibited higher microbial diversity compared to commercial samples, with greater amplicon sequence variant (ASV) richness. Functional bacterial groups correlated with enhanced protein and mineral bioavailability, supporting dawadawa’s role as a probiotic and functional food. Findings highlight the impact of fermentation practices on microbial diversity and nutrient composition, underscoring the importance of preserving traditional methods while optimizing production for food security and sustainability.

## 1. Introduction

Fermented foods have long been an integral part of various cultures worldwide, offering enhanced flavor, extended shelf life, and improved nutritional profiles [1]. In West African countries such as Nigeria and Ghana, one traditional delicacy is dawadawa, a fermented product derived from the seeds of the African locust bean tree (*Parkia biglobosa*) [2, 3]. *Parkia biglobosa* is a deciduous tree in the family Fabaceae that is much valued in Central and West Africa. It is cultivated for its pods containing sweet pulp and nutrient‐dense seeds, which undergo fermentation into condiments including dawadawa, iru, and soumbala. The tree’s seeds, bark, leaves, and roots have established therapeutic benefits, including antidiabetic, wound healing, antioxidant, antibacterial, and analgesic activities, in both traditional and scientific contexts [4]. In Northern Ghana, the major tribal communities that integrate dawadawa as a condiment include Bolgatanga, Wa, Damongo, Tamale, and Bolgatanga [5]. Dawadawa serves as a staple seasoning, imparting a rich umami flavor to dishes such as tuo zaafi, tubaani, and alefu soup, while also providing a significant source of nutrients for local communities [6]. This traditional food product contributes to the region’s culinary identity and significantly supports local economies and sustainable food systems [7]. The fermentation process of locust bean seeds is a meticulous practice that has been refined over centuries. Like many indigenous fermented foods, the production of dawadawa creates valuable employment opportunities and helps reduce unemployment in local communities [7]. It presents opportunities to scale up traditional food processing methods and establish supply chains that contribute to Africa’s food security and economic development [8]. Traditionally, the seeds are boiled, dehulled, and then fermented for several days, during which a diverse array of microorganisms, predominantly bacteria and fungi, act upon them [6, 9, 10]. This microbial activity is crucial, as it not only develops the characteristic flavor and aroma of dawadawa but also enhances its nutritional value [11]. Studies have identified various bacterial strains, including species of *Bacillus*, *Micrococcus*, *Staphylococcus*, and *Lactobacillus*, as dominant microorganisms in fermented locust bean products [3, 11, 12].

Dawadawa’s significance extends beyond its cultural importance. The condiment is particularly valued for its probiotic properties and rich nutritional profile, which includes essential proteins, amino acids, mineral salts, crude fat, and carbohydrates [13, 14]. Research indicates that fermented locust bean seeds contain approximately 35% protein, 29% lipids, and 16% carbohydrates [15]. While previous research has suggested that variations in indigenous ethnic fermentation practices might influence dawadawa’s nutritional profiles, this relationship has not been thoroughly investigated with empirical evidence [16]. Additionally, the fermentation process has been shown to increase the bioavailability of these nutrients while reducing antinutritional factors, thereby enhancing the overall nutritional profile of the product [14].

Earlier microbiological studies of dawadawa primarily relied on culture‐based methods, which have identified various *Bacillus* species (including *B. subtilis*, *B. badius*, *B. licheniformis*, *B. cereus*, and *B. megaterium*) as key fermentation agents [10, 17, 18]. However, these traditional methodologies are limited in their ability to provide a comprehensive understanding of the microbial communities involved in fermentation [19, 20]. Modern culture‐independent approaches, particularly those utilizing bacterial 16S DNA sequencing, have emerged as powerful tools for characterizing microbial communities in fermented foods, offering profound insights into the complex microbial ecosystems present during fermentation [8, 19, 21, 22]. Recent research has begun to explore bacterial communities in fermented African foods, including preliminary studies of dawadawa [23]. However, these investigations have typically focused on single pooled samples, leaving gaps in our understanding of how commercial fermentation processes and unique ethnic practices influence bacterial community composition. This knowledge gap represents a significant opportunity to enhance our understanding of this critical traditional food product [21].

Understanding the microbial diversity and nutritional composition of dawadawa is essential for several reasons. First, it provides insights into the traditional fermentation processes that have been passed down through generations [1, 2, 6, 24, 25]. Second, it highlights the potential health benefits associated with consuming fermented foods rich in probiotics and essential nutrients [3, 8, 12, 11]. Third, it underscores the cultural significance of dawadawa in Northern Ghana, where it remains a dietary staple and a symbol of culinary heritage [6, 23, 26]. This study investigates the microbial communities involved in the fermentation of locust bean seeds, analyzing how different ethnic fermentation methods influence their bacterial composition and nutritional profile. Thus, the research enhances understanding of traditional African fermented foods by comparing commercial and domestic processes to determine their health and nutritional benefits for local communities, as well as identify unique bacterial signature, and proximate compositions, contributing to broader knowledge of indigenous food preservation and enrichment techniques.

## 2. Materials and Methods

### 2.1. Sample Collection and Preparation

Dawadawa samples were collected from specific markets across six major settlements in Ghana: Bolgatanga Central Market (Bolgatanga, Upper East Region), Damongo Township Market (Damongo, Savannah Region), Aboabo Market (Tamale, Northern Region), Navrongo Central Market (Navrongo, Upper East Region), Nana Bosoma Market (Sunyani, Bono Region), and Wa Central Market (Wa, Upper West Region). Samples were categorized as either commercial (from established producers in Tamale and Sunyani markets) or domestic (traditional household production in other locations). All samples were collected in sterile containers (polypropylene containers) (50‐mL falcon tubes, Corning Inc., Corning, New York, United States) and transported under controlled conditions (maintained at 4°C in insulated cooler boxes) to the laboratory within 24 h for analysis. Table 1 shows the local names used for this fermented condiment in each region.

**Table 1:** Local names of fermented locust bean condiment across study regions in Ghana.

**Administrative region**	**Sampling location**	**Market/source**	**Dominant local language**	**Local name**	**Literal translation**	**Sample type**
Upper East	*Bolgatanga*	Bolgatanga Central Market	Frafra/Gurene	Kalgu	*Fermented beans*	Domestic
Upper East	*Navrongo*	Navrongo Central Market	Kasena	Kalgu	*Fermented beans*	Domestic
Savannah	*Damongo*	Damongo Township Market	Gonja	Dawadawa	*Fermented locust beans*	Domestic
Northern	*Tamale*	Aboabo Market	Dagbani	Dawadawa	*Fermented locust beans*	Commercial
Upper West	*Wa*	Wa Central Market	Waali	Kalgu	*Fermented beans*	Domestic
Bono	*Sunyani*	Nana Bosoma Market	Twi/Akan	Dawadawa	*Fermented locust beans*	Commercial

### 2.2. Reagents and Chemicals

All reagents used were of analytical grade. Petroleum ether, concentrated sulfuric acid (H₂SO₄), sodium hydroxide (NaOH), boric acid, hydrochloric acid (HCl), and selenium‐based catalyst were obtained from Sigma‐Aldrich (St. Louis, Missouri, United States). Mixed indicator solution was prepared using methyl red and bromocresol green from Merck KGaA (Darmstadt, Germany). Asbestos fiber was sourced from Fisher Scientific (Hampton, New Hampshire, United States). PowerSoil DNA Isolation Kit was purchased from QIAGEN (Hilden, Germany). PCR reagents and primers for 16S rRNA amplification were obtained from Thermo Fisher Scientific (Waltham, Massachusetts, United States). All solutions were prepared using ultrapure water (18.2 M*Ω*·cm) from a Milli‐Q water purification system (Millipore, Burlington, Massachusetts, United States).

### 2.3. Physicochemical Composition of Dawadawa

The physicochemical composition of dawadawa was determined using established analytical methods [27]. The details of each approach are outlined below.

#### 2.3.1. Moisture Content Determination

Two grams (2 g each) of fermented *Parkia biglobosa* seed (dawadawa) powder was weighed accurately into two previously ignited and weighed crucibles. The crucibles were placed in a thermostatically controlled oven at 1050°C for 5 h, after which they were removed and placed in a desiccator to cool. The crucibles were each weighed intermittently until constant weight was attained, and the moisture content was determined [27]. The moisture content was calculated per Equation (1):

(1) Moisture content %=W2−W3W2−W1×100

*W*_1_ is the weight of empty crucible, *W*_2_ is the weight of empty crucible + sample before heating, and *W*_3_ is the weight of empty crucible + sample after heating.

#### 2.3.2. Ash Value Determination

Two grams (2 g each) of fermented *Parkia biglobosa* seed (dawadawa) powder was weighed accurately into two previously ignited and weighed crucibles and placed in a muffle furnace (preheated at 600°C for 2 h to incinerate). After which, the crucibles were removed and transferred directly to desiccators and allowed to cool. They were each weighed, and the percentage of ash was determined [27]:

(2) Ash content %=W3−W1W2−W1×100

*W*_1_ is the weight of empty crucible, *W*_2_ is the weight of empty crucible + sample before ashing, and *W*_3_ is the weight of empty crucible + sample after ashing.

#### 2.3.3. Fat Content Determination

Two grams (2 g each) of fermented *Parkia biglobosa* seed (dawadawa) powder was weighed accurately into a cellulose thimble. A 250‐mL round‐bottom flask was cleaned, dried, and weighed. An amount of 150‐mL petroleum ether was measured into the round‐bottom flask. The extraction apparatus was set up with the quick‐fit condenser connected to the Soxhlet extractor. The refluxing was done on a heating mantle (low heat) for 16 h. The flask was removed, and the petroleum ether was recovered (evaporated) on a steam bath in a fume chamber. The flask with the extracted fat was dried in an oven at 105°C for 30 min and cooled in a desiccator, after which the flask containing only the extracted fat was weighed. The percentage crude fat was calculated using the formula below:

(3) Fat content %=Wf+f−WfWs×100

*W*_s_ is the weight of the sample, *W*_f_ is the weight of the flask, *W*_f+f_ is the weight of round‐bottom flask + fat, and *W*_f+f_ − *W*_f_ is the weight of fat.

#### 2.3.4. Crude Fiber Determination

Crude fiber is reported as the loss in weight on ignition of dry residue remaining after the sample is digested with 1.25% H_2_SO_4_ and 1.25% NaOH under specific conditions to decarbonize the sample by removing proteins and carbohydrates. About 2 g of the defatted sample was transferred into a 750‐mL Erlenmeyer flask with 0.5 g of asbestos added. Then, 200 mL of boiling 1.25% H_2_SO_4_ was added, and the flask was connected to a cold finger condenser. The setup was placed on a hot plate to boil for 30 min. The flask was removed thereafter and the contents filtered through a linen cloth in a funnel with boiling water until no longer acidic. The char and asbestos were washed back into the flask, and 200 mL of boiling 1.25% NaOH was added, with the flask again connected to the condenser and boiled for 30 min. The content was again filtered through a linen cloth and washed thoroughly with boiling water until it was no longer basic. The residue was transferred into a Gooch crucible and dried in an oven at 100°C for 1 h. The crucible and the content were cooled in a desiccator and then weighed and placed in a preheated electric furnace at 600°C for 30 min. It was cooled and reweighed, and the percentage crude fiber was calculated as follows:

(4) Crude fiber content %=W1−W2W3×100

*W*_1_ is the weight of the crucible + dried sample, *W*_2_ is the weight of the crucible + ashed sample, *W*_3_ is the weight of the sample, and *W*_1_ − *W*_2_ is the weight of the fiber.

#### 2.3.5. Protein Content Determination

This was determined by the Kjeldahl method by Sáez‐Plaza [28], where the total nitrogen was determined and multiplied by a conversion factor of 6.25 to attain the protein content. A 2‐g sample of the fermented *Parkia biglobosa* seed (dawadawa) powder was mixed with a sufficient selenium‐based catalyst plus 25 mL of concentrated H_2_SO_4_ in a digestion flask. The mixture was heated in a digestion rack under a fume chamber until a clear solution was obtained, called the digest. The digest was transferred into a 100‐mL conical flask and diluted with distilled water to the mark and used for the analysis. For distillation, 10 mL of the digest was mixed with 20 mL of 40% NaOH solution in a Kjeldahl distillation apparatus. The mixture was distilled into 25 mL of 20% boric acid containing five drops of mixed indicator. A total of 50‐mL distillate was collected and titrated against 0.1 M HCl solution until a light pink color was obtained (end point). A reagent blank was distilled and titrated. Hence, protein content was calculated using the following formula: blank value = 0.15 mL:

(5) %total nitrogen=1000.01401100+VA−VB×NA××w×10

*V*_A_ is the volume in milliliter of standard acid used in titration, *V*_B_ is the volume in milliliter of standard acid used in blank, *N*_A_ is the normality of acid (HCl), and *W*is the weight in grams of the sample:

(6) %crude protein=%nitrogen×factor

#### 2.3.6. Carbohydrate Determination

Carbohydrate content of the samples was acquired by difference using the derived data and the following equation (Equation (7)):

(7) Carbohydrate %=100 %moisture+%ash+%fat+%protein

### 2.4. Mineral Composition of Dawadawa

The mineral analysis used defatted dawadawa samples processed to eliminate carbohydrates and proteins. Ashed samples were used to characterize the materials. Dawadawa minerals were characterized by Flame Atomic Absorption Spectrometry using a PerkinElmer AAnalyst 400 (PerkinElmer Inc., Waltham, Massachusetts, United States) [29]. The ashed sample was mixed with 5‐mL HCl, diluted with deionized water, and filtered into a 50‐mL volumetric flask. Protocol reagents liberated the components to be determined from the filtrate. The liquid samples were then aspirated into a flame with a beam of light directed through the flames into a monochromatic detector that detects the amount of light absorbed by the atomized element in the flame since each element has its absorption wavelength in the UV‐visible spectrum. Each element’s energy absorbed was computed into its concentration using Beer’s law [30].

### 2.5. DNA Analysis and Sequencing

To analyze the bacterial communities present in dawadawa, we employed a comprehensive approach encompassing DNA extraction, amplification of the 16S rRNA gene’s V3‐V4 hypervariable regions, sequencing, and subsequent bioinformatics analysis.

#### 2.5.1. DNA Extraction

Genomic DNA was extracted from 0.5 g of dawadawa sample using the PowerSoil DNA Isolation Kit (QIAGEN, Hilden, Germany), following the manufacturer’s protocol. This kit is widely utilized for its efficiency in isolating high‐quality DNA from complex food matrices, including fermented products.

#### 2.5.2. PCR Amplification of 16S rRNA Gene

The V3‐V4 hypervariable regions of the 16S rRNA gene were targeted for amplification due to their effectiveness in differentiating bacterial taxa [31]. A two‐step PCR approach was employed: First PCR: Utilizing universal primers specific to the V3‐V4 regions, initial amplification was performed to enrich the target sequences. Second PCR: Indexing primers were used to attach sequencing adapters and unique barcodes to each sample, facilitating multiplexing during sequencing.

#### 2.5.3. Sequencing

The prepared libraries were sequenced on the DNBSEQ‐G400 platform (MGI Tech Co. Ltd., Shenzhen, China), generating paired‐end reads of 2 × 150 bp. This platform is recognized for its high throughput and accuracy in sequencing amplicon libraries.

### 2.6. Statistical Analysis

Statistical analysis was conducted using multiple software platforms to ensure comprehensive data interpretation. For proximate composition and mineral content analysis, descriptive statistics including mean and standard deviation were calculated for all parameters. One‐way analysis of variance (ANOVA) was employed to identify significant differences among samples from different locations, followed by Tukey’s honest significant difference (HSD) post hoc test for pairwise comparisons. A confidence interval of 95% (*p* < 0.05) was set for all statistical tests. These analyses were conducted using IBM SPSS Statistics for Windows, Version 22.0 (IBM Corp., Armonk, New York).

For 16S rRNA gene sequencing data, raw sequencing reads were processed using the DADA2 plugin within the QIIME 2 platform (Version 2023.2) [32, 33]. Quality filtering, chimera removal, and inference of exact amplicon sequence variants (ASVs) were performed to provide high‐resolution bacterial taxa identification. Taxonomic assignment was performed by aligning ASVs against the Greengenes database using a naïve Bayes classifier [34].

Diversity analyses including alpha diversity, beta diversity, and differential abundance analysis were conducted using the Microeco Package in R [35]. Comparison of bacterial communities between samples was performed using the Kruskal–Wallis test in Statistical Analysis of Metagenomic Profiles (STAMP) [36]. Correlation analysis between proximate composition and bacterial communities was explored using canonical correspondence analysis in XLSTAT 2022 software (Addinsoft, Paris, France) while dendrogram clustering was performed using Paleontological Statistics (PAST v4).

The statistical data analysis for the proximate composition, mineral content, and 16S rRNA gene sequencing of dawadawa samples was conducted using established statistical and bioinformatics methodologies.

## 3. Results

### 3.1. Proximate and Mineral Content of Dawadawa Samples

Our results show variations in the proximate composition of fermented locust bean (dawadawa) from different northern communities (*p* ≤ 0.05) (Table 2). The highest fat content was observed in Wa (27.70%) and Tamale (27.65%), while the lowest was recorded in Navrongo (17.45%). This suggests that dawadawa from Wa and Tamale may have a higher lipid contribution, which could influence flavor and energy content. Besides Wa and Tamale, the fat levels in dawadawa varied significantly among the communities. Sunyani had the highest fiber content (7.32%), followed by Wa (7.26%), while Bolgatanga had the lowest (6.39%). Higher fiber content is beneficial for digestion and gut health. Wa exhibited the highest mineral content (9.30%), whereas Tamale had the lowest (3.20%). The differences in ash content suggest variations in mineral composition depending on the location. Navrongo had the highest moisture content (12.00%), indicating a higher water retention capacity. In contrast, Wa had the lowest (8.25%), which could impact shelf life and microbial growth. Apart from Tamale and Navrongo samples, protein levels varied significantly among the communities sampled, ranging between 36.12 ± 0.31 and 50.0 ± 0.13. Sunyani recorded the highest protein content (50%), making its dawadawa a rich protein source. Wa had the lowest protein content (36.12%), suggesting variability in the fermentation process or raw materials used. Nitrogen‐free extract (NFe) levels also ranged between 5.10 ± 0.06 and 16.37 ± 1.03. Bolgatanga had the highest NFe carbohydrate content (16.37%), whereas Tamale had the lowest (5.10%). This suggests that dawadawa from Bolgatanga may serve as a better energy source.

**Table 2:** Proximate composition of dawadawa from different communitie**s.**

**Source**	**Fat (%)**	**Crude fiber (%)**	**Total ash (%)**	**Moisture (%)**	**Protein (%)**	**NFe carbohydrate (%)**
Bolgatanga	22.80 ± 0.70^c^	6.39 ± 0.23^c^	8.30 ± 0.14^b^	8.40 ± 0.28^d^	37.74 ± 0.13^d^	16.37 ± 1.03^a^
Damongo	24.95 ± 0.71^b^	6.64 ± 0.12^bc^	6.20 ± 0.28^c^	8.50 ± 0.70^d^	40.37 ± 0.12^c^	13.35 ± 0.36^b^
Tamale	27.65 ± 0.21^a^	6.84 ± 0.06^b^	3.20 ± 0.14^d^	9.70 ± 0.14^c^	47.51 ± 0.06^b^	5.10 ± 0.06^f^
Navrongo	17.45 ± 0.21^d^	6.62 ± 0.01^bc^	7.95 ± 0.21^b^	12.00 ± 0.28^a^	47.35 ± 0.10^b^	8.64 ± 0.79^d^
Sunyani	18.05 ± 0.07^d^	7.32 ± 0.04^a^	7.20 ± 0.14^c^	10.90 ± 0.42^b^	50.00 ± 0.13^a^	6.53 ± 0.05^e^
Wa	27.70 ± 0.14^a^	7.26 ± 0.02^a^	9.30 ± 0.34^a^	8.25 ± 0.21^d^	36.12 ± 0.31^e^	11.36 ± 0.12^c^

*Note:* Mean levels of proximate components (mean ± standard deviation, *n* = 3) in the same column with different alphabets vary significantly (*p* ≤ 0.05).

Significant differences were observed in proximate composition parameters (moisture, protein, fat, fiber, ash, and carbohydrate content) across the different dawadawa samples. The protein content showed a statistically significant difference (*p* < 0.05), suggesting variations in processing or fermentation. Differences in moisture content also indicated that some samples had significantly lower water activity (*p* < 0.05), which could affect shelf life and microbial growth. The fat content varied significantly across samples, with two showing the highest concentrations (*p* < 0.05). This aligns with previous studies indicating that fermentation and processing techniques impact fat composition [37]. Fiber content was significantly higher in the Sunyani sample (*p* < 0.05), supporting findings by Adedokun et al. [38], who observed similar trends in fermented African locust bean products. The Fe concentration was highest in all samples among the minerals studied and was followed by Zn, Mn, and Cu in descending order, respectively (Table 3). Apart from nitrogen, the levels of other mineral ions such as phosphorus, potassium, calcium, sodium (Na), and magnesium were present in trace amounts (Table 3). Sunyani samples had the highest nitrogen (8.00%), indicating a potentially higher protein content, as reflected in Table 2. The Wa sample had the highest potassium (1.75%), while Sunyani also recorded high potassium levels (1.69%). Phosphorus was highest in Sunyani (0.72%) and lowest in Tamale (0.54%). The Bolga sample had the highest calcium (1.61%), suggesting potential benefits for bone health. Magnesium levels were highest in the Bolga sample (0.57%), followed by the Sunyani and Wa samples (0.31%), whereas Damongo and Navrongo had the lowest levels (0.24%–0.25%). The calcium and magnesium levels significantly differed between the samples (*p* < 0.05), implying processing effects on mineral retention.

**Table 3:** Mineral composition of dawadawa from different communities.

**Source**	**N**	**P**	**K**	**Ca**	**Mg**	**Na**	**Fe**	**Zn**	**Cu**	**Mn**
	**%**						**mg/kg**			
Bolga	6.04 ± 0.02^d^	0.60 ± 0.01^d^	1.55 ± 0.01^e^	1.61 ± 0.01^a^	0.57 ± 0.01^a^	0.09 ± 0.0^a^	135.00 ± 6.51^a^	91.77 ± 2.60^a^	47.49 ± 0.05^a^	70.59 ± 12.54^a^
Damongo	6.46 ± 0.02^c^	0.63 ± 0.01^c^	1.60 ± 0.01^d^	0.87 ± 0.02^d^	0.25 ± 0.01^c^	0.09 ± 0.0^b^	79.60 ± 1.56^e^	49.86 ± 1.73^d^	18.21 ± 0.28^e^	50.60 ± 14.80^b^
Tamale	7.60 ± 0.01^b^	0.54 ± 0.02^e^	1.64 ± 0.02^c^	0.97 ± 0.01^c^	0.29 ± 0.00^b^	0.07 ± 0.0^d^	107.45 ± 4.03^b^	75.58 ± 4.75^b^	29.40 ± 0.40^d^	50.72 ± 15.38^b^
Navrongo	7.58 ± 0.02^b^	0.61 ± 0.02^c^	1.57 ± 0.02^de^	0.73 ± 0.02^e^	0.24 ± 0.01^c^	0.07 ± 0.0^e^	83.90 ± 0.85^d^	40.23 ± 4.36^de^	42.69 ± 0.87^b^	46.05 ± 22.99^b^
Sunyani	8.00 ± 0.02^a^	0.72 ± 0.01^a^	1.69 ± 0.03^b^	0.86 ± 0.03^d^	0.31 ± 0.01^b^	0.08 ± 0.0^c^	111.65 ± 0.35^b^	37.75 ± 4.97^e^	29.51 ± 0.42^d^	44.09 ± 22.42^b^
Wa	5.78 ± 0.05^e^	0.70 ± 0.03^b^	1.75 ± 0.01^a^	1.05 ± 0.02^b^	0.31 ± 0.00^b^	0.09 ± 0.0^b^	91.45 ± 0.50^c^	70.60 ± 1.34^bc^	40.66 ± 0.64^c^	43.63 ± 22.63^b^

*Note:* Mean levels of minerals (mean ± standard deviation, *n* = 3) in the same column with different alphabets vary significantly (*p* < 0.05).

The Na content of the samples varied minimally, ranging from 0.07% to 0.09%. The highest iron concentration was recorded in the Bolga sample (135.00 mg/kg), followed by Sunyani (111.65 mg/kg) and Tamale (107.45 mg/kg). This suggests these sources may be better for preventing iron deficiency anemia. Damongo had the lowest iron content (79.60 mg/kg). Bolga samples contained the highest zinc (91.77 mg/kg), which is essential for immune function. Sunyani had the lowest zinc (37.75 mg/kg). Iron and zinc contents showed staggering variations, but statistical significance was observed (*p* > 0.05), suggesting that bioavailability factors might be at play. Copper was highest in the Bolga sample (47.49 mg/kg) and lowest in the Damongo sample (18.21 mg/kg). Manganese levels were highest in the Bolga sample (70.59 mg/kg) and lowest in the Wa sample (43.63 mg/kg). Significant differences (*p* < 0.05) were observed in most mineral levels across the different communities. Sunyani had the highest overall nitrogen and phosphorus levels, which could indicate a richer nutritional profile. Bolga samples stood out for their high calcium, iron, zinc, and manganese, making them nutritionally beneficial. Wa exhibited the highest potassium, potentially beneficial for cardiovascular health. These differences could be due to variations in soil composition, climate, and processing methods in each region.

### 3.2. Bacterial Community Analysis

#### 3.2.1. Bacteria Sequencing Output From Dawadawa Samples

Partial sequencing of the 16S V3‐V4 hypervariable bacteria region yielded an average of 78,027 reads, and 52,687 were successfully merged reads after denoising and filtering. The species richness diversity of bacteria in dawadawa samples differed significantly (Mann–Whitney test: *p* ≤ 0.05) based on the observed number of ASVs; however, evenness‐based diversity measures (Shannon and Simpson) showed no difference between commercial and domestic samples (Table 4). Commercial samples from urbanized communities such as Tamale and Sunyani had fewer ASVs than domestic samples from Damongo, Navrongo, Wa, and Bolgatanga. The ASV richness and evenness showed that Wa has the least diversity of bacteria, followed by Damongo, Tamale, Sunyani, Navrongo, and Bolgatanga in descending order, respectively.

**Table 4:** Diversity measures of the bacteria communities in dawadawa.

**Location**	**Type**	**Observed**	**Shannon**	**Simpson**	**Fischer**
Wa	Domestic	171^c^	1.89^a^	0.66^a^	12.09^b^
Bolgatanga	Domestic	170^c^	2.99^a^	0.90^a^	11.73^b^
Damongo	Domestic	186^d^	2.38^a^	0.83^a^	14.67^b^
Navrongo	Domestic	205^e^	2.91^a^	0.89^a^	14.54^b^
Tamale	Commercial	148^a^	2.53^a^	0.83^a^	9.74^a^
Sunyani	Commercial	154^b^	2.77^a^	0.90^a^	11.11^a^
*p* value		0.00988	0.7195	0.467	0.012

*Note:* Alpha diversity measures in the same column with different alphabets vary significantly (*p* ≤ 0.05).

For the observed species richness, the highest number of observed bacterial species was in Navrongo (205), while Tamale had the lowest (148) (Table 3). This suggests that domestic dawadawa has greater microbial diversity compared to commercial production. The Shannon index, which measures species diversity, ranged between 1.89 (Wa) and 2.99 (Bolgatanga). The values indicate moderate diversity, with no significant differences among locations (*p* = 0.7195). The Simpson index, on the other hand, measures the dominance of bacteria. Table 3 shows that most locations had high diversity (values close to 1). The lowest value was in Wa (0.66), suggesting lower bacterial diversity, while the highest was in Sunyani (0.90). The Fischer index revealed the highest bacterial diversity was found in Damongo (14.67) and Navrongo (14.54), while Tamale had the lowest (9.74). The *p* value (0.012) indicates significant differences in bacterial diversity across locations.

#### 3.2.2. Bacteria Diversity From Dawadawa Samples

The analysis of bacterial communities in dawadawa showed that Firmicutes is the predominant phylum, followed by Proteobacteria, Actinobacteria, Bacteroidetes, Fusobacteria, and Cyanobacteria in descending order, respectively (Figure 1a). The variation in bacterial phyla among different communities may be due to differences in fermentation conditions, local environmental factors, or starter cultures used. The presence of phylum Firmicutes is significant due to its role in lactic acid fermentation. At the family level, Bacillaceae was the most abundant, followed by Lactobacillaceae, Planococcaceae, Pseudomonadaceae, Staphylococcaceae, Streptococcaceae, Aerococcaceae, Clostridiaceae, Peptostreptococcaceae, and Enterobacteriaceae (Figure 1b).

Figure 1Relative abundance of bacteria (a) phyla and (b) families in dawadawa from different communities.

**Figure:**
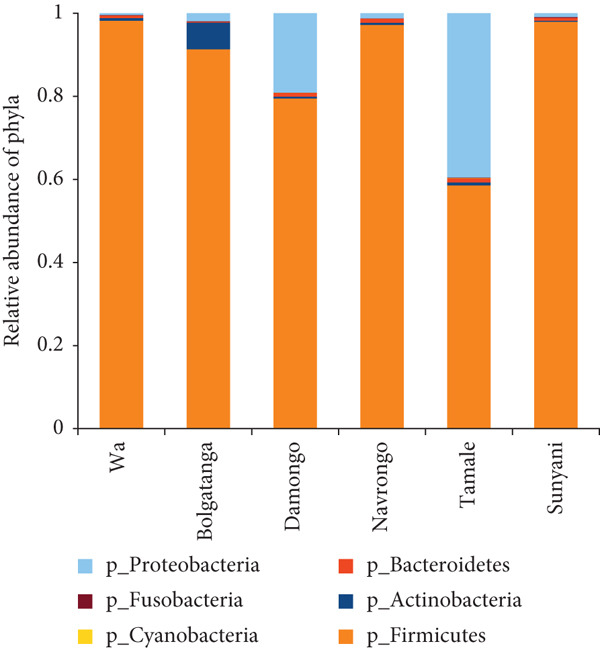
(a)

**Figure:**
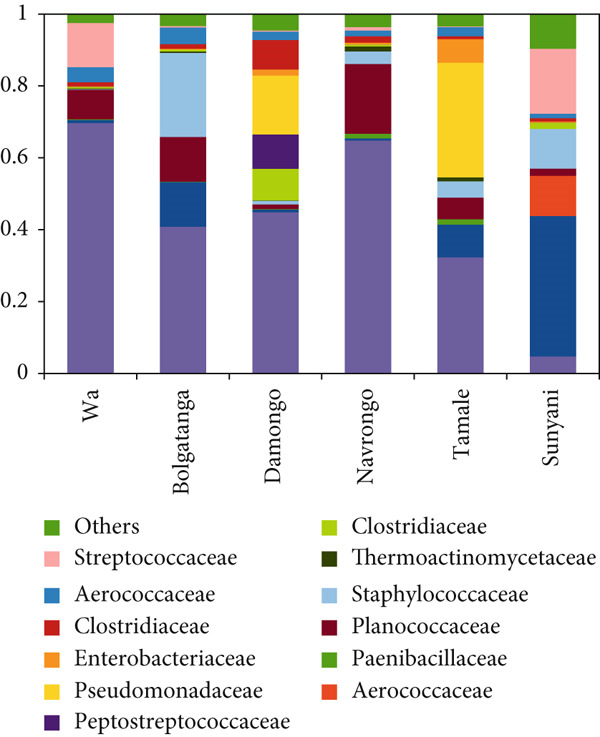
(b)

*Bacillus subtilis* was the predominant *Bacillus* species among the annotated bacteria in commercial and domestic samples (Figures 2a, 2b, 2c, and 2d). Other notable bacteria species in dawadawa samples include *Pseudomonas fragi*, *Clostridium* spp. (*C. cochlearium*, *C. sporogenes*, *C. maritimum*, *C. paraputrificum*, and *C. tetani*), *Macrococcus* spp. (*M. caseolyticus* and *M. brunensis*), *Ureibacillus thermosphaericus*, *Pediococcus acidilactici*, *Streptococcus* spp. (*S. equi*, *S. agalactiae*, and *S. infantarius*), *Staphylococcus* spp. (*S. saprophyticus*, *S. simulans*, and *S. hyicus*), *Lactobacillus* spp. (*L. fermentum* and *L. gasseri*), and *Paraclostridium bifermentans.*

Figure 2Statistical analysis of bacterial families in dawadawa from (a) Sunyani and Tamale, (b) Wa and Sunyani, (c) Wa and Bolgatanga, and (d) Bolgatanga and Tamale.

**Figure:**
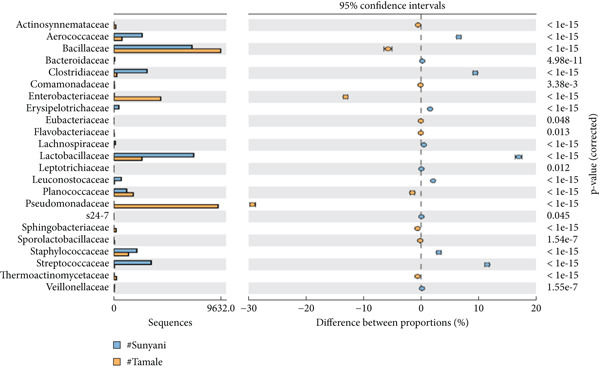
(a)

**Figure:**
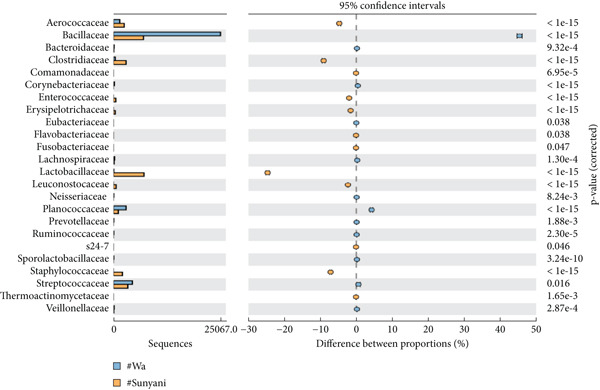
(b)

**Figure:**
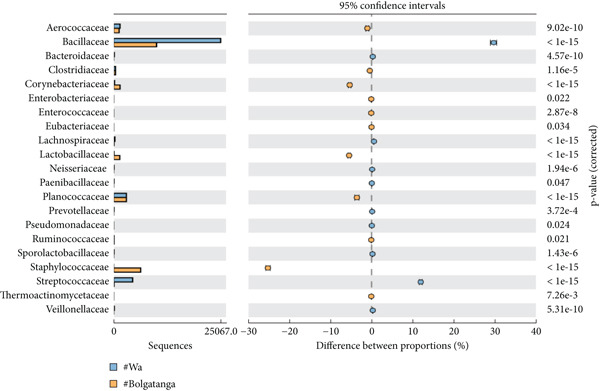
(c)

**Figure:**
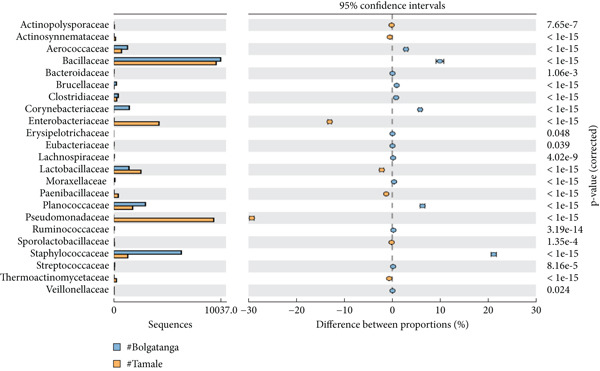
(d)

Beta diversity of bacterial communities in dawadawa explored by PCoA based on Bray–Curtis dissimilarity indicated no significant difference between domestic and commercial samples (*p* value, 0.133). Commercial and domestic samples were succinctly distinguished based on beta diversity and dendrogram clustering of ASVs (Figure 3a). Canonical correlation analysis of differentially abundant bacterial ASVs extracted from LEfSe analysis and proximate components showed two leading associations between bacteria and nutrients/microelements (Figure 3b).

Figure 3(a) Dendrogram showing hierarchical clustering of bacteria communities in dawadawa from different communities and (b) canonical correlation analysis of proximate components and microbiome in dawadawa.

**Figure:**
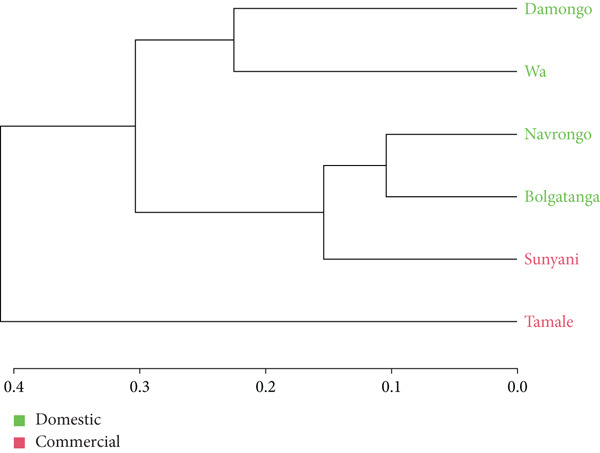
(a)

**Figure:**
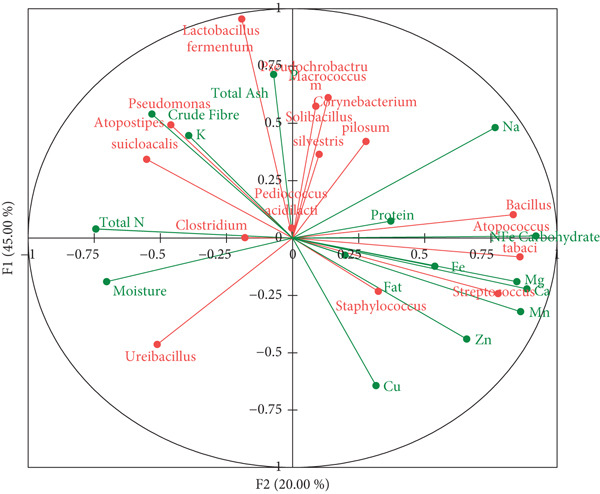
(b)

Specifically, predominant bacterial genera such as *Bacillus*, *Staphylococcus*, *Atopococcus*, and *Streptococcus* were associated with fat, protein, NFE carbohydrate, Fe, Mg, Ca, Mn, and Zn. Total ash, crude fiber, phosphorus, and potassium levels correlated with *Corynebacterium*, *Solibacillus*, *Lactobacillus*, *Macrococcus*, *Pseudomonas*, *Pediococcus*, *Pseudochrobactrum*, and *Pediococcus*. Network analysis of bacterial ASVs showed indirect linkages or associations between predominant genera such as *Bacillus*, *Macrococcus*, *Pseudomonas*, *Clostridium*, and *Staphylococcus* with minor genera. Statistical analysis of commercial samples (Sunyani and Tamale) at the family level revealed significant differences in the abundance of bacterial communities except for Eubacteriaceae and S24‐7 (*p* = 0.05).

## 4. Discussion

### 4.1. Proximate and Mineral Composition of Dawadawa

#### 4.1.1. Proximate Composition of Dawadawa Samples

The levels of fats in dawadawa from the selected northern settlements in Ghana concur with the outcome of earlier research suggesting comparatively higher fat levels than other African fermented seeds or condiments [37, 39–42]. Olasupo et al. [43] attributed the high fat content in dawadawa to the limited lipolytic activities of bacteria, thus leaving behind high proportions of unsaturated fatty acids. In this study, the levels of fat differed significantly between northern communities except for Tamale and Wa samples, which recorded higher fat content, and this arises from different processing steps, indigenous microbiota, and cultivars [2, 25, 44, 45]. Locally, the dehulled seeds are boiled before fermentation, therefore reducing the fat content, but fermentation does not further reduce the fat content as it serves as a substrate for microbial hydrolysis, which contributes to the unique ammoniacal odor [16, 46–48]. Dietary fiber is essential for a good bowel movement and reduces the risks of diabetes, colon cancer, and cardiovascular diseases [8, 42, 49]. The crude fiber content observed in dawadawa from Northern Ghana is higher than most local legumes, which range from 2.1% to 7.1%, and this shows that dawadawa is a good source of dietary fiber [50, 51]. The ash content of samples from Wa is the highest recorded locally, although fermentation of dawadawa reduces the ash content due to microbial metabolism [2, 42, 46, 52]. More so, lower ash content (3.20 ± 0.14) as recorded in dawadawa from Tamale still falls within the range for most legumes of 2% in pea to 5% in soya bean [53, 54]. The ash content reflects the mineral composition, and this reveals comparatively higher levels of minerals in dawadawa than other legume condiments, making it the local condiment of choice [16, 40, 45]. The protein content of dawadawa from the northern communities is consistent with the other research findings, which show that the fermented product contains more proteins than raw unfermented seeds, thereby making it a good source of protein for malnourished children and adults [55, 56]. Although contradicting reports exist regarding the low levels or absence of essential amino acids in dawadawa, other findings have indicated comparatively higher levels of essential amino acids in dawadawa attributed to the activities of extracellular proteases secreted by microbial consortia involved in the fermentation process [40, 45, 57–59]. Apart from Damongo and Bolgatanga, the levels of carbohydrates in dawadawa from the selected communities are lower than most reports, and this is characteristic of local cultivars, which contain low levels of carbohydrates in the raw seeds and pulp [2, 16, 42, 60]. High moisture sustains microbial transformation of protein‐rich legume seeds; however, dawadawa absorbs environmental moisture high in the humid tropical terrain, making it liable to continued fermentation even during storage [61]. The moisture content of dawadawa observed in this study is concomitant with the outcome of earlier studies that recorded a range between 7% and 15% moisture in the final product, and this usually accounts for about 5%–10% increase in the moisture content during fermentation, favoring the activities of *Bacillus* spp. [44, 59, 60, 62]. More so, commercially sourced samples contained lower carbohydrate levels, implicating limited breakdown of complex fibers through limited microbial diversity starter cultures involved in fermentation. Conversely, activities of enzymes (lignin‐modifying enzymes, cellulases, and hemicellulose) secreted by microbial communities result in the partial breakdown of fibers, releasing considerable levels of carbohydrates in the seed of *Parkia biglobosa* after fermentation [5, 59, 63, 64].

#### 4.1.2. Mineral Composition of Dawadawa Samples

The high nitrogen content observed in Sunyani (8.00%) suggests a potentially high protein content. Studies on *Parkia biglobosa* (dawadawa) report protein contents ranging from 35% to 45% on a dry‐weight basis, depending on fermentation and processing methods [7]. The nitrogen levels in this study are within the expected range, reinforcing the traditional role of dawadawa as a protein supplement in West African diets. Phosphorus values (0.54%–0.72%) align with previous findings, where dawadawa phosphorus content typically ranges from 0.5% to 0.8% [24]. Potassium levels (1.55%–1.75%) are slightly higher than those reported in earlier studies, where values ranged between 1.2% and 1.6% [58]. The elevated levels in Wa (1.75%) suggest potential soil‐based influences. The calcium content in Bolga (1.61%) is considerably high, which is consistent with studies by [65], who reported calcium levels of 1.5%–1.7% in traditionally fermented dawadawa. Magnesium values (0.24%–0.31%) match findings from [66, 67], who reported 0.25%–0.35% in Sudanese dawadawa. The slightly lower values in Navrongo and Damongo may be due to soil mineral depletion or differences in fermentation techniques. The high iron levels in Bolga (135 mg/kg) are notable. Existing literature reports iron concentrations in dawadawa ranging from 90 to 140 mg/kg [58]. The Bolga sample falls within this range and suggests its potential role in addressing iron‐deficiency anemia. The copper values (18.21–47.49 mg/kg) align with literature estimates of 20–50 mg/kg in fermented legumes [7]. The relatively lower levels in Damongo (18.21 mg/kg) may indicate variability in soil micronutrient composition. Manganese levels (43.63–70.59 mg/kg) are within expected values for fermented African locust beans (50–80 mg/kg) [68]. Zinc levels (37.75–91.77 mg/kg) are generally higher than those found in previous studies (50–80 mg/kg) [43, 48]. The high zinc in Bolga suggests improved immune‐supportive benefits. Specifically, the levels of Fe, Zn, Cu, and Mn in dawadawa are comparatively higher than most studies in the subregion, and this arises from the local varieties and ethnic practices utilized in fermentation [5, 59]. The relatively high levels of these trace elements (Fe, Cu, Zn, and Mn) in dawadawa could suggest potential health benefits such as promoting hematopoiesis, neutralization of free radicals, nutrient metabolism, insulin secretion, and alleviating metabolic disorders [69–72]. The low levels of mineral ions such as Na, Mg, K, and P are consistent with few research findings; however, most studies have reported higher levels of these mineral salts [57, 63, 73]. This inconsistency primarily arises from prolonged soaking, boiling, and uncontrolled fermentation, which cause the leaching of nutrients into the aqueous phase and increased microbial acquisition, thereby reducing the minerals content significantly [52, 69, 74, 75].

### 4.2. Nutritional Implications of Bacterial Communities

The analysis of bacterial communities of dawadawa in this study revealed complex relationships between microbial activity and nutritional composition, with two distinct bacterial–nutrient associations emerging from the data. The first significant pattern centered on primary nutrient transformation, where predominant bacterial genera, including *Bacillus*, *Staphylococcus*, and *Streptococcus*, demonstrated strong correlations with major nutritional components [24, 58]. Through their metabolic activities, these bacteria exhibited substantial influence over the protein, fat, and carbohydrate profiles [48]. Their presence and abundance strongly suggested a crucial role in enhancing nutrient bioavailability, effectively transforming the raw ingredients into a more nutritionally accessible form [68]. A second, equally important association emerged regarding prebiotic–probiotic interactions within the fermented product. Lactic acid bacteria (LAB) showed robust correlations with fiber content, suggesting an intricate relationship between these microorganisms and the substrate’s structural components [76]. This association provides compelling evidence for the prebiotic nature of dawadawa’s fiber components, which appear to support the growth and activity of beneficial bacteria [77]. The symbiotic relationship between the fiber content and LAB further supports dawadawa’s potential role in promoting gut health, as these interactions contribute to a healthy gastrointestinal environment [78]. This finding adds another dimension to understanding dawadawa’s nutritional value, suggesting benefits beyond its essential nutrient content.

### 4.3. Microbial Diversity Patterns in Commercial Versus Domestic Samples

Analysis of bacterial communities revealed intriguing differences between domestic and commercial dawadawa samples, with domestically fermented products showing distinctly higher numbers of ASVs [24, 68]. This increased microbial diversity in domestic samples can be primarily attributed to traditional processing methods employed in household production [76]. The manual manipulation during dehulling and molding processes, combined with the use of traditional wrapping materials such as banana and plantain leaves, introduces a rich variety of environmental microflora [48, 79]. Additionally, the use of traditional fermentation vessels like calabash contributes to this diverse microbial ecosystem, as these natural materials harbor their own unique microorganisms [1]. In contrast, commercial production methods demonstrate notably different characteristics that influence microbial diversity [77]. These facilities employ more controlled fermentation conditions and significantly reduced manual handling, leading to a more standardized but less diverse microbial community [26]. The use of standardized starter cultures and consistent processing environments, while ensuring product uniformity, appears to limit the introduction of diverse environmental microorganisms that are characteristic of traditional processing methods [80]. Firmicutes emerged as the predominant bacterial phylum throughout all samples, comprising between 74% and 91% of the total bacterial population [78]. This dominance aligns well with their established role in alkaline fermentation processes and their significant contribution to developing the unique flavor compounds that characterize dawadawa [1, 7]. The observed variation in Firmicutes proportions between domestic and commercial samples provides compelling evidence of how different fermentation practices influence the final product’s microbial composition [58]. While commercial samples showed more consistent but lower Firmicutes levels, domestic samples exhibited more significant variation but generally higher proportions, reflecting the impact of traditional versus controlled fermentation methods [80].

### 4.4. Role of *Bacillus* Species in Fermentation

The identification of several novel *Bacillus* species in dawadawa has significantly expanded our understanding of the fermentation process. Our study revealed the presence of previously unreported species, including *B. acidicola*, *B. sanguinis*, *B. alkalinitrilicus*, *B. thermotolerans*, *B. coagulans*, and *B. koreensis*, each contributing unique characteristics to the fermentation process [1, 24]. These findings provide new insights into the complexity of traditional fermentation and its impact on the final product’s qualities [48, 58].

The metabolic contributions of these *Bacillus* species play a crucial role in transforming raw ingredients. The *B. subtilis* group, in particular, exhibits peak proteolytic activity after 72 h of fermentation, leading to the release of peptides and free amino acids from proteins [77, 79]. This process is accompanied by significant ammonia production, which creates the characteristic alkaline conditions essential for proper fermentation [78, 80]. Additionally, these bacteria facilitate early‐stage carbohydrate breakdown through their amylase activity, contributing to the development of the product’s distinctive properties [26, 68]. Each newly identified species appears to serve specific functions that enhance the fermentation process and final product quality. *B. alkalinitrilicus* contributes to product preservation through its unique ability to hydrolyze nitriles into amides [81]. *B. koreensis* produces maltooligosaccharides, which enhance the moisturizing properties of the final product while potentially supporting the growth of beneficial gut bacteria [1]. *B. thermotolerans* adds another dimension to the fermentation process through its generation of novel antibacterial metabolites, which may contribute to both product preservation and potential health benefits [82]. These diverse functions highlight the sophisticated microbial ecosystem that develops during traditional dawadawa fermentation [80].

### 4.5. Health‐Promoting Properties

Our investigation revealed multiple health‐promoting properties of dawadawa, with significant implications for its role in both nutrition and functional food applications [24, 26]. The probiotic potential of dawadawa emerges as a particularly noteworthy aspect, characterized by the presence of diverse LAB, which were found in notably higher abundance in domestic samples [58, 78]. The identification of beneficial species such as *Lactobacillus fermentum* and *Pediococcus acidilactici* suggests that dawadawa could serve as an effective vehicle for delivering probiotic benefits to consumers [68, 80]. The nutritional profile of dawadawa further enhances its health‐promoting qualities. Analysis revealed remarkably high protein content, ranging from 36% to 50%, making it a valuable protein source in local diets [48, 79]. The product also demonstrates an impressive essential mineral composition, particularly rich in iron, zinc, copper, and manganese [76, 77]. These minerals, coupled with prebiotic fiber components, contribute significantly to its nutritional value and potential health benefits [1]. The presence of these prebiotic elements suggests a capacity to support beneficial gut microbiota, enhancing the product’s overall health impact [78, 81]. Beyond basic nutrition, dawadawa exhibits several functional properties that contribute to its health‐promoting potential. Various *Bacillus* species present in the fermented product produce antimicrobial compounds, which may offer protective benefits [26, 82]. The interaction between fiber components and microbes appears to support gut health, creating a synergistic effect that extends beyond simple nutritional value [80]. Furthermore, the microbial transformation processes during fermentation enhance nutrient bioavailability, potentially improving the body’s absorption and utilization of nutrients [7, 68]. These combined properties position dawadawa as not just a traditional condiment but a functional food with significant potential health benefits [1].

### 4.6. Implications for Production and Food Security

Our findings reveal significant implications for the future of both traditional and commercial dawadawa production, particularly in the context of food security and sustainable development [24, 26, 83]. The comprehensive understanding of key bacterial communities gained through this research provides valuable insights for optimizing production methods [58, 84]. This knowledge could inform the development of more effective starter cultures that better replicate the benefits of traditional fermentation while maintaining product consistency [80, 85]. Moreover, the research opens possibilities for standardizing traditional practices in a way that preserves the crucial microbial diversity while ensuring product safety and quality [77, 86]. The study’s findings also underscore dawadawa’s important contribution to food security in local communities [87]. Its high nutritional value and sustainable production methods make it an invaluable resource for addressing nutritional needs in resource‐limited areas [68, 78, 88]. The traditional production methods, passed down through generations, represent not just a means of food preparation but also embody important cultural knowledge that merits preservation and documentation [1, 76, 89] Perhaps most significantly, the identification of unique bacterial signatures across different settlements demonstrates how local practices profoundly influence the final product’s characteristics [1, 48]. This variation highlights the delicate balance between preserving traditional knowledge and optimizing production methods [80, 84]. As we move forward with production improvements, it becomes crucial to maintain these distinct characteristics that arise from local practices while enhancing the product’s probiotic properties and overall quality [7, 26, 90]. This balance between innovation and tradition could serve as a model for developing other traditional fermented foods, ultimately contributing to both food security and cultural preservation [82].

## 5. Conclusion

This study provides a comprehensive analysis of dawadawa’s microbial diversity, nutritional composition, and potential health benefits. The results confirm significant variations in proximate and mineral content across different locations, with Sunyani samples exhibiting the highest protein concentration. The microbial community analysis revealed *Bacillus* species as dominant fermentative agents, with domestic dawadawa demonstrating greater bacterial diversity than commercial products. The presence of probiotic LAB and mineral‐rich compositions underscores dawadawa’s functional food potential, supporting gut health and addressing micronutrient deficiencies. The observed microbial–nutrient interactions further highlight the crucial role of fermentation in enhancing nutrient bioavailability. Importantly, the study emphasizes the need to balance traditional fermentation methods with modern food safety and standardization approaches. The findings suggest that optimizing fermentation while preserving microbial diversity could enhance dawadawa’s nutritional benefits and economic value. Future research should explore targeted starter cultures and controlled fermentation conditions to improve consistency without compromising indigenous microbial ecosystems. By advancing the understanding of dawadawa’s microbiome and nutritional attributes, this study contributes to its sustainable production, reinforcing its role in food security and cultural preservation in Northern Ghana and beyond.

## Conflicts of Interest

The authors declare no conflicts of interest.

## Funding

No funding was received for this manuscript.

## Data Availability

The data that support the findings of this study are available from the corresponding author upon reasonable request.
